# HIV Drug Resistance Surveillance in Honduras after a Decade of Widespread Antiretroviral Therapy

**DOI:** 10.1371/journal.pone.0142604

**Published:** 2015-11-11

**Authors:** Santiago Avila-Ríos, Claudia García-Morales, Daniela Tapia-Trejo, Rita I. Meza, Sandra M. Nuñez, Leda Parham, Norma A. Flores, Diana Valladares, Luisa M. Pineda, Dixiana Flores, Roxana Motiño, Víctor Umanzor, Candy Carbajal, Wendy Murillo, Ivette Lorenzana, Elsa Y. Palou, Gustavo Reyes-Terán

**Affiliations:** 1 Centre for Research in Infectious Diseases, National Institute of Respiratory Diseases, Mexico City, Mexico; 2 HIV National Programme and National Laboratory, Honduran Ministry of Health, Tegucigalpa, Honduras; 3 Instituto Nacional Cardio Pulmonar, Tegucigalpa, Honduras; 4 Hospital Mario Catarino Rivas, San Pedro Sula, Honduras; 5 Unidad de Salud Metropolitana, La Ceiba, Honduras; 6 Hospital del Sur, Choluteca, Honduras; 7 Universidad Nacional Autónoma de Honduras, Tegucigalpa, Honduras; 8 Hospital Escuela Universitario, Tegucigalpa, Honduras; University of British Columbia, CANADA

## Abstract

**Introduction:**

We assessed HIV drug resistance (DR) in individuals failing ART (acquired DR, ADR) and in ART-naïve individuals (pre-ART DR, PDR) in Honduras, after 10 years of widespread availability of ART.

**Methods:**

365 HIV-infected, ART-naïve, and 381 ART-experienced Honduran individuals were enrolled in 5 reference centres in Tegucigalpa, San Pedro Sula, La Ceiba, and Choluteca between April 2013 and April 2015. Plasma HIV protease-RT sequences were obtained. HIVDR was assessed using the WHO HIVDR mutation list and the Stanford algorithm. Recently infected (RI) individuals were identified using a multi-assay algorithm.

**Results:**

PDR to any ARV drug was 11.5% (95% CI 8.4–15.2%). NNRTI PDR prevalence (8.2%) was higher than NRTI (2.2%) and PI (1.9%, p<0.0001). No significant trends in time were observed when comparing 2013 and 2014, when using a moving average approach along the study period or when comparing individuals with >500 vs. <350 CD4+ T cells/μL. PDR in recently infected individuals was 13.6%, showing no significant difference with PDR in individuals with longstanding infection (10.7%). The most prevalent PDR mutations were M46IL (1.4%), T215 revertants (0.5%), and K103NS (5.5%). The overall ADR prevalence in individuals with <48 months on ART was 87.8% and for the ≥48 months on ART group 81.3%. ADR to three drug families increased in individuals with longer time on ART (p = 0.0343). M184V and K103N were the most frequent ADR mutations. PDR mutation frequency correlated with ADR mutation frequency for PI and NNRTI (p<0.01), but not for NRTI. Clusters of viruses were observed suggesting transmission of HIVDR both from ART-experienced to ART-naïve individuals and between ART-naïve individuals.

**Conclusions:**

The global PDR prevalence in Honduras remains at the intermediate level, after 10 years of widespread availability of ART. Evidence of ADR influencing the presence of PDR was observed by phylogenetic analyses and ADR/PDR mutation frequency correlations.

## Introduction

The recent expansion of antiretroviral treatment (ART) coverage in middle/low-income countries has been associated with increasing prevalence of HIV carrying antiretroviral (ARV) drug resistance (DR) mutations in ART-naïve individuals, referred to as “transmitted drug resistance” (TDR). Latin America is a region with high ART coverage, with 45% of the 1.6 million people estimated to live with HIV in the region on ART [1]. A recent comprehensive meta-analysis on HIV TDR, performed from 2000 to 2013 in the Latin America and Caribbean (LAC) region, found an overall TDR level of 7.6%, observing a significant temporal increase in non-nucleoside reverse transcriptase inhibitor (NNRTI) TDR in the study period [2]. This observation agrees with a previous WHO study reporting increasing NNRTI TDR in low- and middle-income countries [3]. Other recent systematic reviews have also shown moderate TDR levels in the LAC region, intermediate between North America/Western Europe and Africa/Southeast Asia [4, 5]. Nevertheless, significant differences on TDR prevalence and patterns are expected to exist in the different countries of the LAC region due to profound differences in HIV management. Thus, continuous HIVDR surveillance is key to maximizing the long-term effectiveness of ART regimens and to ensure the sustainability of ART programmes in each country. In an effort to standardize HIVDR surveillance globally and to increase survey national representativity, the WHO has proposed a comprehensive strategy including 4 protocols to assess HIVDR: TDR in recently infected individuals, TDR in adult individuals starting ART (pre-ART HIVDR, PDR), acquired DR (ADR) in individuals on ART and TDR in children <18 months of age (initial DR, IDR), to be implemented in conjunction with an updated strategy for monitoring early warning indicators for HIVDR [6]. Concept notes are now available for the PDR and ADR protocols [7, 8].

It is estimated that 24,000 (20,000–30,000) persons live with HIV in Honduras, in a concentrated epidemic (mainly in men who have sex with men [MSM], female sex workers [FSW] and garifunas [descendants of West African, Central African, Island Carib, and Arawak people]) with 0.5% prevalence in the general population [9]. Nearly 60% of infected adults are male with a high proportion of heterosexual transmissions [9, 10]. The Honduran HIV/AIDS Programme initiated ART access scale-up in 2002 [11] and the country adopted national guidelines on ART for adults and children in 2003. A significant increase in the number of persons under ART has been observed after more than a decade of the implementation of the national ART scale-up programme, and it is estimated that approximately 40% of all HIV-infected individuals were receiving ART by 2014 [10]. ART is distributed through 49 integral attention centres around the country. Nevertheless, due to extensive under-reporting and lack of well-structured national databases, the cascade of the continuum of care in Honduras is difficult to assess. Although high retention rates on ART at 12 months (85%) have been reported [1], important issues associated with HIVDR are known to occur in HIV management in Honduras, including frequent ARV drug shortages, lack of reagents or personnel for performing follow-up tests, re-selling of ARV drugs by patients who decide not to take them, widespread use of standard first-line ART regimens based on low genetic barrier NNRTI with no access to HIVDR testing, and long time waits to second line ART scheme [5, 12].

A study performed in 2002–2003, including individuals from Tegucigalpa and San Pedro Sula (n = 336), reported an overall TDR prevalence of 9.2% [13]. A later study performed in 2004–2007 in individuals from Tegucigalpa and San Pedro Sula (n = 200) reported a TDR prevalence of 7.0% to any ARV drug [14]. This study also reported high-level TDR in recently infected individuals (n = 24, 20.8%), suggesting a possible increasing trend of global TDR in Honduras, mainly due to an increase in NNRTI TDR, which is consistent with the wide use of this ARV drug family in first line ART regimens. In another recent survey performed between 2004 and 2007 assessing ADR in Honduran individuals from Tegucigalpa and San Pedro Sula with treatment failure after >6 months on ART, drug-resistant HIV was detected in 74% of participants, with high prevalence of multi-class ADR [11]. These observations warrant periodical HIVDR surveys in Honduras, which could inform public health policy making in the country regarding management of HIV infected individuals under ART and the choice of first line ARV regimens.

We present a national study to assess the prevalence and trends of HIVDR in individuals failing ART (ADR) and in ART-naïve individuals (PDR) in Honduras, after more than a decade of widespread availability of ART.

## Methods

### Ethics Statement

This study was evaluated and approved by the Ethics Committees of the National Institute of Respiratory Diseases (INER) in Mexico (E06-09), the Honduran National Ministry of Health, and the National Autonomous University of Honduras, and was conducted according to the principles of the Declaration of Helsinki. All the participants gave written informed consent before blood sample donation.

### Patients

A total of 365 HIV-infected, ART-naïve, and 381 ART-experienced Honduran individuals were enrolled in 5 reference centres in Tegucigalpa: Hospital Escuela Universitario (HEU) and Instituto Nacional Cardio-Pulmonar (INCP); San Pedro Sula: Hospital Mario Catarino Rivas (HMCR); Choluteca: Hospital del Sur (HS); and La Ceiba: Unidad de Salud Metropolitana (USM), between April 2013 and April 2015. For the ART-naïve group, both recently diagnosed and follow-up individuals without ART were included. No individuals with known previous exposure to ARV drugs were included in the study. For the ART-experienced group, individuals showing detectable plasma viral load (>1000 copies/mL) after at least 9 months on ART were included. All individuals fulfilling the previously mentioned criteria were given the choice to enrol in the present study at each of the participating centres. After giving written, informed consent, participating individuals donated a single peripheral blood sample, collected in EDTA Vacutainer tubes (BD, San Jose, CA) for molecular assays and Cyto-Chex BCT tubes (Streck, Omaha, NE) for flow-cytometry assays. Demographic data was collected through direct application of a questionnaire at the time of sample donation. Blood samples were sent to and processed at the Centre for Research in Infectious Diseases (CIENI) of the INER in Mexico City within the following 48 hours of collection. Plasma viral load assays, CD4+ T cell counts and HIV genotyping and HIVDR analyses were performed for each patient. Results were sent back to the Honduran centres for patient follow up.

### HIV Plasma Viral Load and CD4+T Cell Count Estimations

HIV plasma viral load was determined with the m2000 system (Abbott, Abbott Park, IL). CD4+ T cell counts were assessed using the Trucount Kit in FACSCanto II instruments (BD Biosciences, San Jose, CA). Both tests are carried out routinely for patient follow up at the INER.

### HIV Sequencing

Plasma HIV protease-RT sequences were obtained using an in-house-developed protocol with a 3730xl Genetic Analyser instrument (Life Technologies, Carlsbad, CA), as previously reported [15]. The CIENI, INER is a WHO-certified laboratory belonging to the HIVDR Network, fulfilling procedural and infrastructure requirements for HIV genotyping quality assurance and participating in annual proficiency panels for external quality control assessment.

### Genotypic Antiretroviral Drug Resistance Testing

PDR was assessed by the presence of any of the mutations included in the list for HIV TDR surveillance defined and periodically updated by the WHO [16]. Additionally, PDR and ADR analyses were carried out with the Stanford HIV Drug Resistance Database algorithm (v7.0) [17, 18], using the HIVdb programme available on line [19, 20]. To define the presence of ARV drug resistance, a total drug penalty score of 15 or higher to any ARV drug (at least low-level resistance) was considered.

### HIV Subtyping

HIV subtyping was carried out using the sequenced PR-RT segment, with REGA HIV subtyping tool [21]. All recombinants and non-B subtypes were confirmed using the RIP tool [22] and with phylogenetic analyses as explained below.

### Phylogenetic Analyses

Maximum likelihood trees were constructed using MEGA6 [23]. Genetic distances were estimated using the General Time Reversible + Γ + I model using a partial deletion of gaps/missing data treatment. Drug resistance sites and positions with less than 95% site coverage were eliminated from the alignment. Ambiguous bases were allowed at any position. Significance was tested with 1000 bootstrap replicates. Reference sequences for different subtypes, available at the Los Alamos HIV Sequence Database [24] were included.

### Assessment of Recent Infection

Recently infected individuals were identified using a previously described multi-assay algorithm including incidence tests that minimizes false recency results [25]. Briefly, the BED HIV-1 Incidence EIA (Sedia, Portland, OR) was applied to all individuals with CD4+ T cell counts >200 cells/μL and <1 year of HIV diagnosis. Specimens with ODn ≤1.0 were subjected to the confirmatory HIV-1 Lag-Avidity EIA (Sedia). Specimens with avidity index <80% and plasma viral load (pVL) >400 RNA copies/ml were considered as recent seroconversion cases. This multi-assay algorithm significantly reduced the false-recent misclassification rate (only 0.4% individuals infected for >1 year misclassified as recently infected), with a window period (mean duration during which the algorithm classifies a person as recently infected) of 141 days (95% CI, 94–150) [25].

### Statistical Analyses

Group comparisons for clinical and demographic variables were performed using Mann-Whitney U and Fisher’s exact tests for continuous and categorical variables. Pearson correlations were calculated for correlations between PDR and ADR mutation frequencies. All analyses were conducted in GraphPad Prism v6.0 (GraphPad Software, La Jolla, CA) [26].

## Results

### Characteristics of the Study Cohorts

HIV *pol* sequences were obtained for 365 recently diagnosed and follow-up ART-naïve Honduran individuals enrolled at 5 centres located in Tegucigalpa, San Pedro Sula, La Ceiba and Choluteca for the PDR survey (S1 Dataset). The majority of participants were male (59%), with median age at enrolment of 34 years (IQR 26–43), median CD4+ T cell count of 270 cells/μl, (IQR 89–486) and median pVL of 4.6 log RNA copies/ml (IQR 3.9–5.2). Additionally, 381 adult individuals (18 years and older) under ART showing detectable pVL after at least 9 months under ART were enrolled at the same centres for the ADR survey (S1 Dataset). Half of the participants of the ADR study were male, with a median age of 39 years (IQR 33–46), median CD4 T cell count of 160 cells/μl (IQR 64–289) and median pVL of 4.2 log RNA copies/ml (IQR 3.5–4.9). The median time under ART was 80 months (IQR 46–108). In general, nearly 60% of participants in both surveys were single, approximately half had low literacy levels (primary school), and nearly 60% were unemployed (Table 1). The most common risk factor for HIV acquisition was heterosexual. Individuals under ART with detectable pVL were older than ART-naïve individuals; had lower CD4 T cell counts and lower pVL (p>0.01) (Table 1).

**Table 1:** Demographic characteristics of HIV-1-infected Honduran individuals included in the PDR and ADR surveys.

	ART-naïve individuals without PDR (n = 323)		Individuals with PDR (n = 42)		Individuals under ART with detectable pVL (n = 381)	
**Gender [n (%)]**						
Male	187	(57.9)	27	(64.3)	190	(49.9)
Female	136	(42.1)	15	(35.7)	191	(50.1)
**Age [years; median (IQR)]** *	34	(26, 43)	31	(24, 38)	39	(33, 46)
**CD4 T Cell Count [cells/μL; median (IQR)]** *	268	(90, 483)	300	(72, 516)	160	(64, 289)
**Plasma Viral Load [log copies/mL; median (IQR)]** *	4.6	(3.8, 5.2)	4.9	(4.0, 5.4)	4.2	(3.5, 4.9)
**Marital Status [n(%)]**						
Domestic Partnership	93	(28.8)	12	(28.6)	90	(23.6)
Married	32	(9.9)	4	(9.5)	55	(14.4)
Single	180	(55.7)	25	(59.5)	215	(56.4)
Other	11	(3.4)	0	(0.0)	15	(3.9)
Unknown	7	(2.2)	1	(2.4)	6	(1.6)
**Literacy [n (%)]**						
Primary	152	(47.1)	21	(50.0)	218	(57.2)
High School	98	(30.3)	11	(26.2)	99	(26.0)
Degree/Technical	36	(11.1)	5	(11.9)	19	(5.0)
Cannot Read-Write/None	34	(10.5)	5	(11.9)	40	(10.5)
Unknown	3	(0.9)	0	(0.0)	5	(1.3)
**Employment [n (%)]**						
Employed	143	(44.3)	12	(28.6)	158	(41.5)
Unemployed	156	(48.3)	26	(61.9)	212	(55.6)
Student	17	(5.3)	2	(4.8)	7	(1.8)
Unknown	7	(2.2)	2	(4.8)	4	(1.0)
**HIV Risk Factor [n (%)]**						
MSM	44	(13.6)	9	(21.4)	22	(5.8)
Heterosexual	255	(78.9)	29	(69.0)	339	(89.0)
Blood Transfusion	2	(0.6)	2	(4.8)	4	(1.0)
Heterosexual/MSM	10	(3.1)	1	(2.4)	6	(1.6)
PWID	5	(1.5)	0	(0.0)	6	(1.6)
Other	3	(0.9)	0	(0.0)	0	(0.0)
Unknown	4	(1.2)	1	(2.4)	4	(1.0)
**Collection Center [n (%)]**						
INCP	64	(19.8)	9	(21.4)	111	(29.1)
HEU	33	(10.2)	4	(9.5)	58	(15.2)
HMCR	114	(35.3)	14	(33.3)	121	(31.8)
USM**	68	(21.1)	12	(28.6)	45	(11.8)
HS	44	(13.6)	3	(7.1)	43	(11.3)
Unknown	0	(0.0)	0	(0.0)	3	(0.8)

*Difference between PDR/ART-exposed, and ART-naïve/ART-exposed, (p<0.01), Mann-Whitney test

**Difference between PDR/ART-exposed, and ART-naïve/ART-exposed, (p<0.01), Fisher test; PDR, Pre-ART Drug Resistance; ADR, Acquired Drug Resistance; IQR, Inter Quartile Range; MSM, men who have sex with men; PWID, people who inject drugs; INCP, Instituto Nacional Cardio-Pulmonar (Tegucigalpa); HEU, Hospital Escuela Universitario (Tegucigalpa); HMCR, Hospital Mario Catarino Rivas (San Pedro Sula); USM, Unidad de Salud Metropolitana (La Ceiba); HS, Hospital del Sur (Choluteca).

Considering only the PR-RT region, HIV subtype B was highly prevalent in the study population: 98.9% for all the participants included both in the PDR and the ADR surveys (n = 744, data for two individuals not available). Only 1.1% of viruses were classified as non-B subtypes or recombinants: 0.7% were BD unique recombinant forms, 0.1% CRF12_BF, 0.1% A1 and 0.1% C.

### PDR Prevalence, Patterns and Trends

The overall PDR prevalence for the complete 2-year study period (April 2013-April 2015) was 11.5% (95% CI 8.4–15.2%), based on the WHO HIVDR mutation list (Table 2). PDR was higher to NNRTI (8.2%) than to NRTI (2.2%) or PI (1.9%) (p<0.01; Table 2). Nevertheless, using the Stanford score HIVDR definition, PDR to any ARV drug was 16.4% (95% CI 12.8–20.6), mainly associated with NNRTI PDR (12.9%). This difference was mostly due to the presence of polymorphic mutations at RT position E138 (in nearly 5% of the circulating viruses), associated with reduced susceptibility to rilpivirine and etravirine and not considered by the WHO TDR surveillance mutation list. No demographic/clinical variables were associated with risk of developing PDR (Table 1).

**Table 2:** PDR in a Honduran HIV-1-infected cohort, April 2013-April 2015 (n = 365).

	WHO Mutation List ^a^				Stanford Score≥15 ^b^			
	n	(%)	[95% CI]		n	(%)	[95% CI]	
Any ARV Drug	42	(11.5)	[8.4,	15.2]	60	(16.4)	[12.8,	20.6]
NNRTI	30	(8.2)	[5.6,	11.5]	47	(12.9)	[9.6,	16.8]
NRTI	8	(2.2)	[1.0,	4.3]	6	(1.6)	[0.6,	3.5]
PI	7	(1.9)	[0.8,	3.9]	10	(2.7)	[1.3,	5.0]

^a^ Pre-Antiretroviral Treatment Drug Resistance (PDR) estimated using the WHO HIV transmitted drug resistance surveillance mutation list.

^b^ PDR estimated with the Stanford algorithm (v7.0), with a threshold of ≥15 for at least one antiretroviral drug of the specified class. ARV, Antiretroviral; NNRTI, Non-Nucleoside Reverse Transcriptase Inhibitors; NRTI, Nucleoside Reverse Transcriptase Inhibitors; PI, Protease Inhibitors.

In order to look for possible PDR trends in time, we compared the PDR prevalence observed during the first (April 2013-March 2014, n = 189) vs. the second (April 2014-March 2015, n = 176) year of the study. No significant trends in time were observed when comparing the two years (S1 Table), nor when using a moving average approach along the study period (S1 Fig). We then compared PDR in individuals with >500 CD4 T cells/μL vs. <350 CD4 T cells/μL as the first group would be expected to be enriched in individuals at early chronic stage, while the later group in individuals with advanced infection. Comparison of these two groups did not show significant differences in PDR prevalence either: 14.1% vs. 11.7% to any ARV drug respectively (S2 Table). Further exploring PDR changes in time, TDR in recently infected individuals was assessed. Using a multi-test algorithm including CD4+ T cell counts, pVL and two recency tests (see Methods), 103 individuals (28%) were found to be recently infected, showing a TDR level of 13.6% (95% CI 7.6–21.8%). No significant differences were observed between TDR levels in recently infected individuals and PDR in individuals with longstanding infection (S2 Table). Taken together, these observations suggest an intermediate PDR prevalence in the country with no significant evidence of recent increasing or decreasing trends.

The most prevalent PDR mutations were M46IL (1.4%) to PI, T215 revertants (0.5%) to NRTI, and K103NS (5.6%) to NNRTI. E138GAR mutations, conferring DR to rilpivirine and etravirine were also highly prevalent (4.7%), causing higher PDR estimations when using the Stanford DR definition (Table 3).

**Table 3:** Frequency of pre-ART and acquired HIV drug resistance mutations in Honduras April 2013-April 2015.

	All ART-naïve, n (%) (n = 365)		Individuals with PDR, n (%) (n = 42) ^a^		ART-experienced ≤48 months, n (%) (n = 98)		ART-experienced >48 months, n (%) (n = 283)	
	Protease Inhibitors (PI)							
L10F	1	(0.3)	0	(0.0)	0	(0.0)	13	(4.6)
V11IL	0	(0.0)	0	(0.0)	0	(0.0)	2	(0.7)
K20IT	3	(0.8)	1	(14.3)	0	(0.0)	5	(1.8)
L23I	0	(0.0)	0	(0.0)	0	(0.0)	1	(0.4)
L24I	0	(0.0)	0	(0.0)	0	(0.0)	5	(1.8)
D30N	0	(0.0)	0	(0.0)	2	(2.0)	0	(0.0)
V32I	0	(0.0)	0	(0.0)	1	(1.0)	1	(0.4)
L33F	3	(0.8)	0	(0.0)	1	(1.0)	9	(3.2)
E35G	1	(0.3)	0	(0.0)	0	(0.0)	0	(0.0)
K43T	0	(0.0)	0	(0.0)	0	(0.0)	5	(1.8)
M46IL	5	(1.4)	5	(71.4)	3	(3.1)	19	(6.7)
I47V	1	(0.3)	1	(14.3)	1	(1.0)	3	(1.1)
I50V	0	(0.0)	0	(0.0)	0	(0.0)	3	(1.1)
F53L	0	(0.0)	0	(0.0)	0	(0.0)	1	(0.4)
I54VA	0	(0.0)	0	(0.0)	0	(0.0)	17	(6.0)
I54M	0	(0.0)	0	(0.0)	1	(1.0)	0	(0.0)
Q58E	2	(0.5)	2	(28.6)	2	(2.0)	10	(3.5)
G73CSTA	0	(0.0)	0	(0.0)	0	(0.0)	4	(1.4)
T74S	3	(0.8)	0	(0.0)	1	(1.0)	2	(0.7)
L76V	0	(0.0)	0	(0.0)	0	(0.0)	4	(1.4)
V82A	0	(0.0)	0	(0.0)	0	(0.0)	10	(3.5)
V82F	0	(0.0)	0	(0.0)	0	(0.0)	4	(1.4)
V82T	0	(0.0)	0	(0.0)	0	(0.0)	1	(0.4)
V82S	0	(0.0)	0	(0.0)	0	(0.0)	1	(0.4)
V82C	0	(0.0)	0	(0.0)	0	(0.0)	4	(1.4)
N83D	0	(0.0)	0	(0.0)	0	(0.0)	1	(0.4)
I84VAC	0	(0.0)	0	(0.0)	0	(0.0)	4	(1.4)
N88D	0	(0.0)	0	(0.0)	1	(1.0)	1	(0.4)
L90M	1	(0.3)	1	(14.3)	0	(0.0)	9	(3.2)
	Nucleoside Reverse Transcriptase Inhibitors (NRTI)							
M41L	0	(0.0)	0	(0.0)	24	(24.5)	65	(23.0)
A62V	1	(0.3)	0	(0.0)	6	(6.1)	3	(1.1)
K65N	0	(0.0)	0	(0.0)	0	(0.0)	1	(0.4)
K65R	0	(0.0)	0	(0.0)	3	(3.1)	4	(1.4)
D67HT	1	(0.3)	1	(14.3)	0	(0.0)	1	(0.4)
D67NG	0	(0.0)	0	(0.0)	22	(22.4)	50	(17.7)
D67E	1	(0.3)	1	(14.3)	1	(1.0)	0	(0.0)
T69D	1	(0.3)	1	(14.3)	1	(1.0)	8	(2.8)
T69NG	0	(0.0)	0	(0.0)	4	(4.1)	21	(7.4)
K70NT	0	(0.0)	0	(0.0)	1	(1.0)	5	(1.8)
K70R	0	(0.0)	0	(0.0)	10	(10.2)	43	(15.2)
K70E	0	(0.0)	0	(0.0)	0	(0.0)	4	(1.4)
L74I	0	(0.0)	0	(0.0)	6	(6.1)	28	(9.9)
L74V	0	(0.0)	0	(0.0)	4	(4.1)	10	(3.5)
V75L	1	(0.3)	1	(14.3)	0	(0.0)	5	(1.8)
V75I	0	(0.0)	0	(0.0)	2	(2.0)	11	(3.9)
V75M	0	(0.0)	0	(0.0)	1	(1.0)	4	(1.4)
F77L	0	(0.0)	0	(0.0)	0	(0.0)	3	(1.1)
Y115F	0	(0.0)	0	(0.0)	1	(1.0)	7	(2.5)
F116Y	0	(0.0)	0	(0.0)	0	(0.0)	1	(0.4)
Q151M	0	(0.0)	0	(0.0)	0	(0.0)	1	(0.4)
M184VI	0	(0.0)	0	(0.0)	70	(71.4)	192	(67.8)
L210W	1	(0.3)	1	(14.3)	10	(10.2)	36	(12.7)
T215Y	0	(0.0)	0	(0.0)	25	(25.5)	63	(22.3)
T215F	0	(0.0)	0	(0.0)	14	(14.3)	34	(12.0)
T215CDESIVA	2	(0.8)	2	(28.6)	4	(4.1)	9	(3.2)
K219QEN	1	(0.3)	1	(14.3)	14	(14.3)	51	(18.0)
K219R	1	(0.3)	1	(14.3)	2	(2.0)	2	(0.7)
	Non Nucleoside Reverse Transcriptase Inhibitors (NNRTI)							
A98G	0	(0.0)	0	(0.0)	3	(3.1)	25	(8.8)
L100I	0	(0.0)	0	(0.0)	5	(5.1)	7	(2.5)
K101HV	0	(0.0)	0	(0.0)	6	(6.1)	5	(1.8)
K101E	4	(1.1)	4	(13.3)	8	(8.2)	24	(8.5)
K101P	0	(0.0)	0	(0.0)	2	(2.0)	10	(3.5)
K103NS	20	(5.5)	20	(66.7)	49	(50.0)	118	(41.7)
K103R	0	(0.0)	0	(0.0)	1	(1.0)	8	(2.8)
V106A	1	(0.3)	1	(3.3)	0	(0.0)	6	(2.1)
V106M	1	(0.3)	1	(3.3)	2	(2.0)	3	(1.1)
V108I	3	(0.8)	0	(0.0)	14	(14.3)	37	(13.1)
E138KQ	1	(0.3)	0	(0.0)	1	(1.0)	1	(0.4)
E138GAR	17	(4.7)	4	(13.3)	2	(2.0)	9	(3.2)
V179AT	6	(1.6)	1	(3.3)	0	(0.0)	8	(2.8)
V179D	14	(3.8)	4	(13.3)	6	(6.1)	37	(13.1)
V179E	0	(0.0)	0	(0.0)	5	(5.1)	13	(4.6)
Y181C	1	(0.3)	1	(3.3)	10	(10.2)	20	(7.1)
Y188L	0	(0.0)	0	(0.0)	5	(5.1)	21	(7.4)
Y188H	0	(0.0)	0	(0.0)	1	(1.0)	1	(0.4)
Y188F	0	(0.0)	0	(0.0)	0	(0.0)	1	(0.4)
Y188C	0	(0.0)	0	(0.0)	0	(0.0)	1	(0.4)
G190S	0	(0.0)	0	(0.0)	11	(11.2)	17	(6.0)
G190A	3	(0.8)	3	(10.0)	12	(12.2)	42	(14.8)
G190E	0	(0.0)	0	(0.0)	2	(2.0)	1	(0.4)
G190CQ	0	(0.0)	0	(0.0)	1	(1.0)	2	(0.7)
H221Y	1	(0.3)	0	(0.0)	8	(8.2)	19	(6.7)
P225H	4	(1.1)	4	(13.3)	13	(13.3)	50	(17.7)
F227L	0	(0.0)	0	(0.0)	1	(1.0)	3	(1.1)
M230L	0	(0.0)	0	(0.0)	7	(7.1)	7	(2.5)
K238N	0	(0.0)	0	(0.0)	0	(0.0)	2	(0.7)
K238T	0	(0.0)	0	(0.0)	3	(3.1)	12	(4.2)
Y318F	1	(0.3)	1	(3.3)	2	(2.0)	3	(1.1)

^a^ Frequency in individuals with pre-ART drug resistance (PDR; defined with the WHO list of mutations for HIV drug resistance surveillance) to the corresponding drug class (PI, n = 7; NRTI, n = 7; NNRTI, n = 30). Mutations that contribute with drug resistance penalty scores in the Stanford algorithm are shown. Only mutations found in the cohort are shown. Mutations considered for the analysis are as follows

NRTIs: M41L, A62V, K65R, D67T, D67H, D67N, D67G, D67E, T69A, T69D, T69ins, T69N, T69C, T69I, T69G, T69S, K70G, K70Q, K70N, K70R, K70E, L74I, L74V, V75L, V75I, V75A, V75T, V75S, V75M, F77L, Y115F, F116Y, V118I, Q151M, M184VI, L210W, T215Y, T215A, T215F, T215CDESIV, K219QEN, K219R.

NNRTIs: V90I, A98G, L100I, K101E, K101P, K103NS, V106A, V106M, V108I, E138KQ, E138GAR, V179AT, V179D, V179E, V179L, V179F, Y181IV, Y181C, Y188L, Y188H, Y188C, G190S, G190A, G190E, G190C, P225H, F227L, M230L, K238T, Y318F.

PIs: L10F, K20I, L23I, L24I, D30N, V32I, L33F, E35G, K43T, M46IL, I47A, I47V, G48VM, I50L, I50V, F53L, F53Y, I54VA, I54L, I54M, I54ST, Q58E, G73CSTA, T74S, L76V, V82A, V82F, V82T, V82S, V82M, V82C, V82L, N83D, I84VAC, I85V, N88D, N88S, L90M.

**Fig 1:**
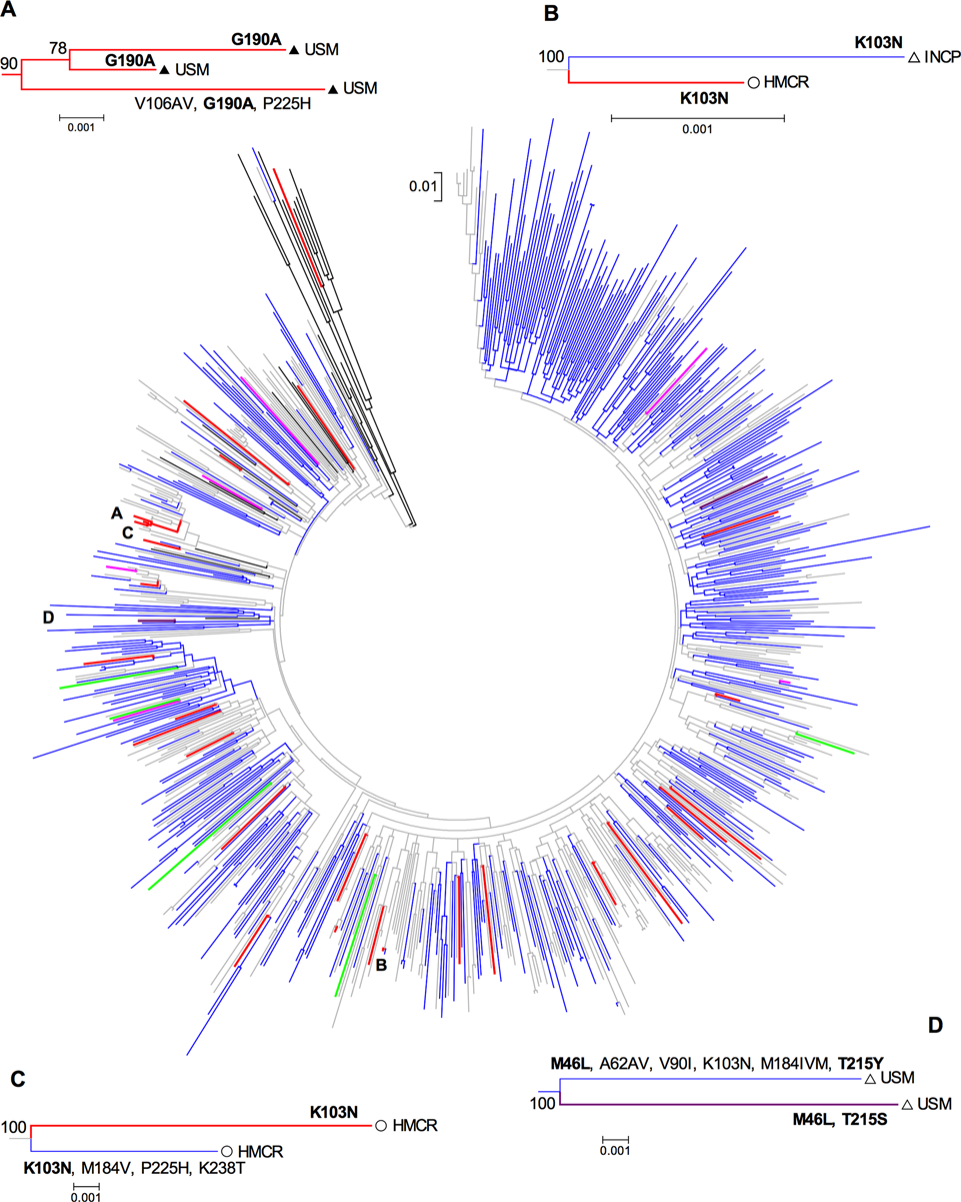
Phylogenetic relations between HIV sequences from ART-naïve and ART-experienced Honduran individuals. A Maximum Likelihood tree including HIV PR-RT sequences from 365 ART-naïve and 381 ART-experienced patients was built, using the General Time Reversible + Γ + I model to estimate genetic distances, with a gamma parameter of 0.4389 estimated for the dataset and 1000 bootstrap repetitions to assess significance. Drug resistance mutation sites as well as positions with less than 95% site coverage were eliminated from the alignment, with a total of 1162 positions included in the final dataset. Branch lengths are measured in number of substitutions per site. All analyses were conducted in MEGA6. Sequences from ART-naïve individuals are shown in grey and sequences from ART-experienced individuals in blue. Sequences with pre-ART drug resistance (PDR) to protease inhibitors (PI, pink), nucleoside RT inhibitors (NRTIs, green), non-nucleoside RT Inhibitors (NNRTIs, red), and more than one ARV family (purple) are coloured. B and non-B reference sequences (shown in black) were obtained from the Los Alamos HIV Database. A-D Clusters of viruses with PDR and bootstrap support >75% are amplified. HIVDR mutations present in the viruses at the tips are shown. Empty triangle, heterosexual male; full-triangle, men who have sex with men; empty circle, female; ART, antiretroviral treatment; USM, Unidad de Salud Metropolitana (La Ceiba); HMCR, Hospital Mario Catarino Rivas (San Pedro Sula); INCP, Instituto Nacional Cardio Pulmonar (Tegucigalpa).

A cluster of viruses with NNRTI PDR from MSM enrolled at La Ceiba was observed. All the viruses in the cluster carried the G190A mutation (Fig 1A). Additionally, 3 clusters including viruses from individuals with ADR and individuals with PDR were observed. The first case included a virus with acquired K103N from a heterosexual male enrolled at Tegucigalpa and a female with transmitted K103N from San Pedro Sula (Fig 1B); the second case included two females enrolled at San Pedro Sula, one with acquired K103N and the other with transmitted K103N (Fig 1C); and the third case included two closely related, multidrug resistant viruses from heterosexual males enrolled at La Ceiba, one containing acquired M46L and T215Y and the other containing transmitted M46 and T215S (Fig 1D).

**Fig 2:**
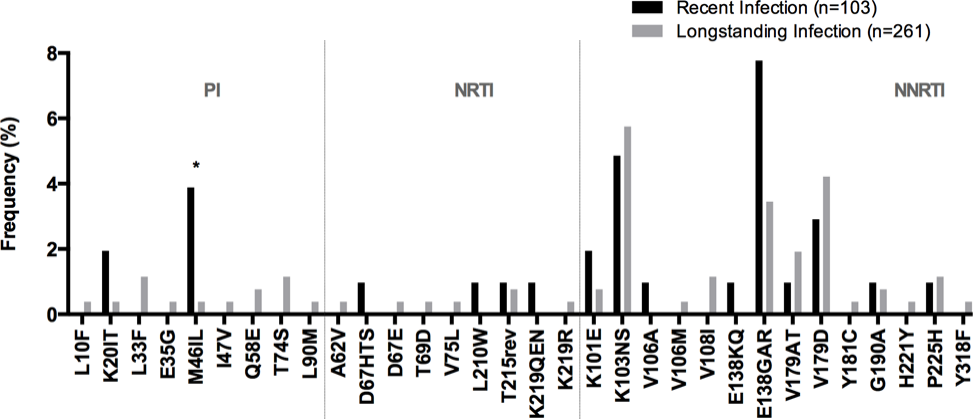
HIVDR mutation frequency comparison in individuals with recent and longstanding infection. Recently infected individuals were identified using a multi-assay algorithm as described in Methods. Only mutations present in any of the comparison groups are shown. Mutations considered for the analysis include WHO TDR surveillance mutations as well as mutations contributing with penalty scores in the Stanford algorithm. For a comprehensive list of mutations considered refer to Table 4. NRTI, Nucleoside RT Inhibitors; NNRTI, Non-nucleoside RT Inhibitors; PI, protease inhibitors; * p<0.05 Fisher’s exact test.

To further explore possible changes of PDR mutational patterns in time, we compared HIVDR mutation frequency in individuals with recent and longstanding infection. In general, HIVDR mutation frequency was similar in the two groups. Only M46IL was more frequent in recently infected individuals (p = 0.0240, Fig 2).

**Table 4:** ADR in Honduran individuals under ART.

ADR ^a^	Individuals with <48 months under ART (n = 98)				Individuals with ≥48 months under ART (n = 283)				p value ^b^
	n	(%)	[95% CI]		n	(%)	[95% CI]		
Any ARV Drug	86	(87.8)	[79.6,	93.5]	230	(81.3)	[76.2,	85.6]	NS
NNRTI	81	(82.7)	[73.7,	89.6]	216	(76.3)	[70.9,	81.2]	NS
NRTI	71	(72.4)	[62.5,	81.0]	195	(68.9)	[63.2,	74.3]	NS
PI	6	(6.1)	[2.3,	12.9]	88	(10.6)	[7.3,	14.8]	NS
1 Drug Family	17	(17.3)	[10.4,	26.3]	44	(15.5)	[11.5,	20.3]	NS
2 Drug Families	67	(68.4)	[58.2,	77.4]	163	(57.6)	[51.6,	63.4]	p<0.0001
3 Drug Families	2	(2.0)	[0.2,	7.2]	23	(8.1)	[5.2,	11.9]	p = 0.0343

^a^ Acquired Drug Resistance (ADR) estimated with the Stanford algorithm (v7.0), with a threshold of ≥15 for at least one antiretroviral drug of the specified class.

^b^ Estimated with Fisher’s exact test. ARV, Antiretroviral; NNRTI, Non-Nucleoside Reverse Transcriptase Inhibitors; NRTI, Nucleoside Reverse Transcriptase Inhibitors; PI, Protease Inhibitors; NS, Not significant (p>0.05).

### ADR Prevalence and Patterns

In order to assess ADR in the study cohort in two different time points, the cohort was divided in individuals who had been on ART for <48 months (n = 98) and for ≥48 months (n = 283). The early time point considering individuals on ART for 12 (±3) months recommended by the WHO ADR surveillance protocol was not applied due to the low number of individuals fulfilling this criterion (n = 15 for the whole study period). The overall ADR prevalence for individuals with <48 months on ART was 87.8% and for the ≥48 months on ART group 81.3% (Table 4). In both cases, PI ADR was lower, compared with NRTI and NNRTI (p<0.0001). No differences were found between ADR levels in the two time points. ADR to two drug families was 68.4% and 57.6% for the <48 and ≥48 months groups respectively (p<0.0001), and ADR to three drug families 2.0 and 8.1% (p = 0.0343, Table 4). Individuals with ≥48 months under ART had and OR of 4.2 (95% CI 1.0–18.4) of presenting ADR to three drug classes compared to individuals with <48 months under ART. M184V (71.4%, <48 months; 67.8%, ≥48 months), and K103N (50.0%, <48 months; 41.7%, ≥48 months) were the most frequent ADR mutations (Table 3).

**Fig 3:**
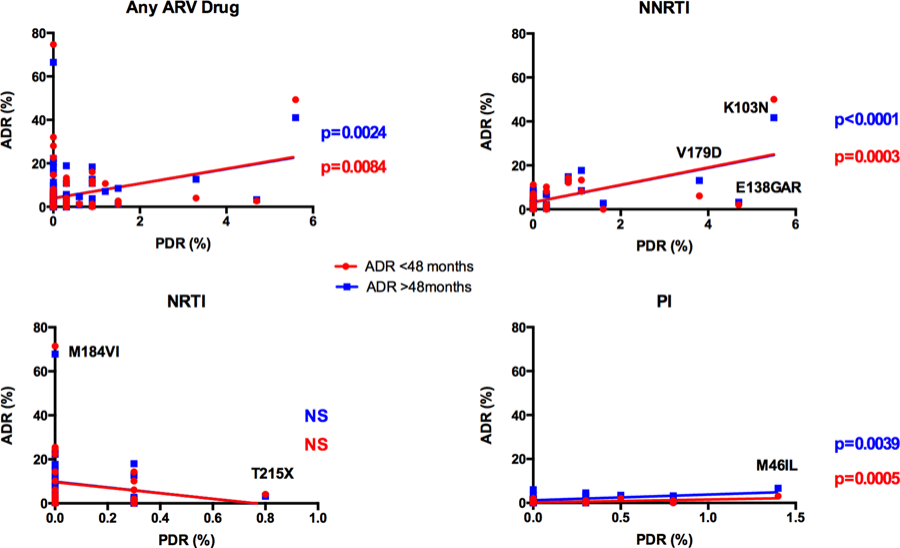
Correlations between PDR and ADR mutation frequency in Honduras. Pearson correlation coefficients were calculated for PDR mutation frequency vs. ADR mutation frequency at <48 and ≥48 months on ART, for the whole study period, for all DR mutations together and dividing them into ARV families. Each point represents one mutation. Some of the most relevant DR mutations are shown. PDR, pre-antiretroviral treatment drug resistance; ADR, acquired drug resistance; NRTI, Nucleoside RT Inhibitors; NNRTI, Non-nucleoside RT Inhibitors; PI, protease inhibitors.

Interestingly, PDR mutation frequency correlated with ADR mutation frequency both in individuals with <48 and ≥48 months on ART for PI and NNRTI (p<0.01 in all cases), but not for NRTI (Fig 3). The lack of correlation for NRTI PDR and ADR remained even after excluding M184V from the analysis. This is in agreement with the observation of various clusters including both individuals with ADR and PDR (Fig 1).

## Discussion

The present work describes PDR and ADR in Honduras from April 2013 to April 2015. This survey results from a logistic effort of the National HIV Programme in Honduras and the National Institute of Respiratory Diseases in Mexico City, an accredited laboratory of the WHO HIVDR Network. The centres that participated in the present study were located in the capital cities of the three departments reporting the highest number of HIV infections in the country, together encompassing more than 75% of all the registered cases of infection: Cortés (with HMCR in San Pedro Sula), Francisco Morazán (with INCP and HEU in Tegucigalpa), and Atlántida (with USM in La Ceiba). Additionally, Choluteca (with HS in Choluteca city) is the sixth department contributing to the total number of infections after Yoro and Colón [27]. Although sampling for the present study was not designed according to the standardized WHO HIV PDR protocol [7], the inclusion of the largest centres, providing service for the most affected urban centres in Honduras makes results relevant at the national level. Indeed, the distribution of individuals from the participating centres in the current PDR survey reflected the national distribution of patients starting ART at each centre: 35% from HMCR (accounting for 43% of patients starting ART in the country), 20% from INCP (18% of patients starting ART), 10% from HEU (16% of patients starting ART), 22% from USM (14% of patients starting ART), and 13% from HS (8% of patients starting ART). Taken together, the 5 participating centres represent nearly 50% of the total number of individuals starting ART in the country (4524/9226 individuals; data from the Honduras National HIV Programme, unpublished). Thus, it can be argued that our study is highly representative of the national Honduran epidemic.

**Fig 4:**
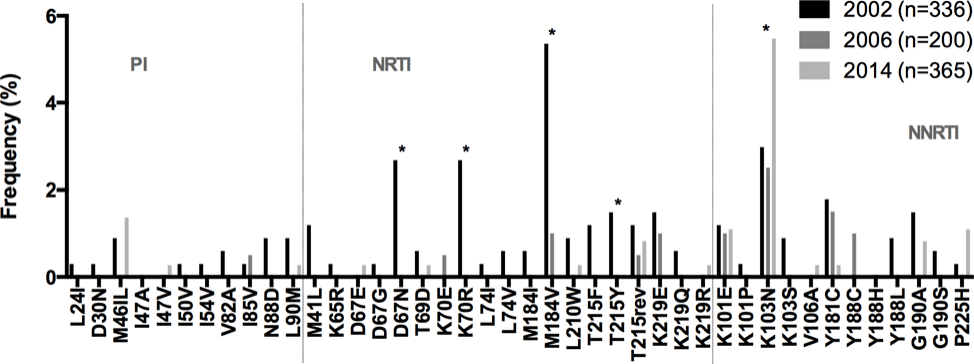
HIVDR mutation frequency in Honduras meta-analysis 2002–20015. HIVDR mutation frequency was compared using data from two previously published studies: Lloyd et al. (median sampling year 2002) [13], and Murillo et al. (median sampling year 2006) [14]; and the present study (median sampling year 2014). Only mutations present in any of the comparison groups are shown. Mutations considered for the analysis include only WHO TDR surveillance mutations. NRTI, Nucleoside RT Inhibitors; NNRTI, Non-nucleoside RT Inhibitors; PI, protease inhibitors. * p<0.05 Fisher’s exact test.

The overall PDR level in Honduras both in 2013 and 2014 remained at the intermediate level, according to WHO thresholds (5–15%) [6], and no significant increasing or decreasing trends were observed in time. This was true when analysing by year of enrolment, by CD4+ T cell count or even when dividing individuals with recent and longstanding infection. Nevertheless, when compared with previous studies using similar criteria to define PDR, the present study revealed interesting tendencies. Lloyd et al., in a study including 336 individuals from Tegucigalpa and San Pedro Sula carried out from 2002–2003, reported an overall PDR prevalence of 9.2% with higher NNRTI (7.1%) and NRTI (7.7%) PDR compared to PI PDR (2.7%) in the beginning of the national ART programme [13]. Later, Murillo et al., in a survey including 200 individuals from Tegucigalpa and San Pedro Sula carried out from 2004 to 2007, observed an overall PDR prevalence of 7.0%, with higher NNRTI (5.0%) and NRTI (3.0%) PDR compared to PI PDR (0.5%) [14]. In the present study, carried out from 2013 to 2015, after more than 10 years of widespread ART rollout, an overall PDR prevalence of 11.5% was observed, with higher NNRTI (8.2%) compared to NNRTI (2.2%) and PI (1.9%) PDR. We performed a meta-analysis, using data from the surveys by Lloyd et al., Murillo et al., and ours, comparing HIVDR mutation frequency. We observed a significant decrease in the frequency of NRTI PDR mutations (D67N, K70R, M184V, T215Y) and an increase in the frequency of K103N (Fig 4), consistent with a long-term decrease in NRTI PDR and increase in NNRTI PDR in Honduras since the beginning of the national ART programme. This observation is in agreement with recent comprehensive studies reporting increasing NNRTI PDR levels in the Latin American and Caribbean region [2, 4].

As expected, ADR was high in non-responding ART-exposed individuals. Moreover, as time on ART increased, the risk of developing DR to more ARV drug families also increased, consistent with the use of PI as a third family of ARV drugs in second line ART schemes. Interestingly, we have shown evidence of the association between ADR and PDR, with a significant correlation between ADR and PDR mutation frequency. This observation is supported by the detection of clusters of sequences including individuals with ADR and PDR. The fact that this correlation was not evident for NRTI could be due to the frequent reversion of mutations with high fitness costs such as M184V upon transmission, while PI and NNRTI mutations replacement rates are low [28]. Indeed, we observed two clusters containing viruses with ADR and PDR in which the virus with ADR possessed M184V and the virus with PDR did not, suggesting reversion upon transmission. However, even when excluding M184V from the analysis, the lack of correlation between NRTI PDR and ADR mutation frequency remained. Indeed, other NRTI DR mutations as M41L, D67N, K70R, L210W, and T215Y/F. Transmission of RT K103N was observed in two cases, while transmission of PR M46L and RT T215 revertants was observed in another case. This result strongly supports recent observations evidencing the common and stable transmission of some TDR mutations including PR L90M and RT K103N, and T215 revertants [29]. Interestingly, transmission characteristics seemed to be varied, including men and women, sometimes from the same geographical area and other times from geographically widespread areas. Importantly, we observed a unique PDR transmission cluster including only MSM from the same centre, suggesting transmission of PDR mutations among ART-naïve individuals. These observations suggest that transmission of HIVDR in Honduras is being driven both by ART-experienced and ART-naïve individuals, and that the demographic characteristics of the individuals involved in these transmissions could be varied depending on the situation, as has also been suggested recently in studies in developed countries [30]. Specifically, transmission from non-responding ART-experienced individuals to ART-naïve individuals seemed to be mostly heterosexual, while transmission among ART-naïve individuals could be more frequent between MSM. Further studies will be needed to assess demographic, host and viral factors influencing HIVDR transmission in Honduras.

## Conclusions

In all, the global PDR prevalence in Honduras remains at the intermediate level after more than 10 years of widespread availability of ART, with NNRTI being the most affected ARV drug family. Although no increasing or decreasing PDR trends in time were observed in the present study, comparisons with previously published studies suggest a long-term increase in NNRTI PDR and decrease in NRTI PDR, consistent with observations in other developing countries. Interestingly, PDR mutation frequency was highly influenced by ADR mutation frequency and evidence of transmission of DR viruses both from non-responding ART-experienced to ART-naïve individuals and among ART-naïve individuals was observed. These observations warrant further HIVDR surveillance studies with high national representativity.

## Supporting Information

S1 Dataset Fasta sequences for all viruses included in the study. (TXT)

S1 Fig HIV PDR trends in Honduras April 2013–April 2015. PDR trends by date of enrolment were estimated using a moving average approach, with 4-month windows, moving by 1-month intervals. PDR prevalence and 95% confidence intervals (CI) are shown. The number of individuals contributed by each participating centre for each time window is also shown. PDR, Pre-antiretroviral Treatment Drug Resistance; NRTI, Nucleoside RT Inhibitors; NNRTI, Non-nucleoside RT Inhibitors; PI, protease inhibitors; INCP, Instituto Nacional Cardio Pulmonar (Tegucigalpa); HEU, Hospital Escuela Universitario (Tegucigalpa); HMCR, Hospital Mario Catarino Rivas (San Pedro Sula); USM, Unidad de Salud Metropolitana (La Ceiba); HS, Hospital del Sur (Choluteca).
(TIFF)

S1 Table PDR in a Honduran HIV-1-infected cohort by year: 2013–2015. (DOC)

S2 Table PDR in a Honduran HIV-1-infected cohort in recently infected individuals and according to CD4+ T cell counts. (DOC)
