# Supplementary figures and images for: HIV Drug Resistance Surveillance in Honduras after a Decade of Widespread Antiretroviral Therapy
Source: PLoS One. 2015 Nov 11;10(11):e0142604. doi: 10.1371/journal.pone.0142604 (PMC4641727; doi:10.1371/journal.pone.0142604)

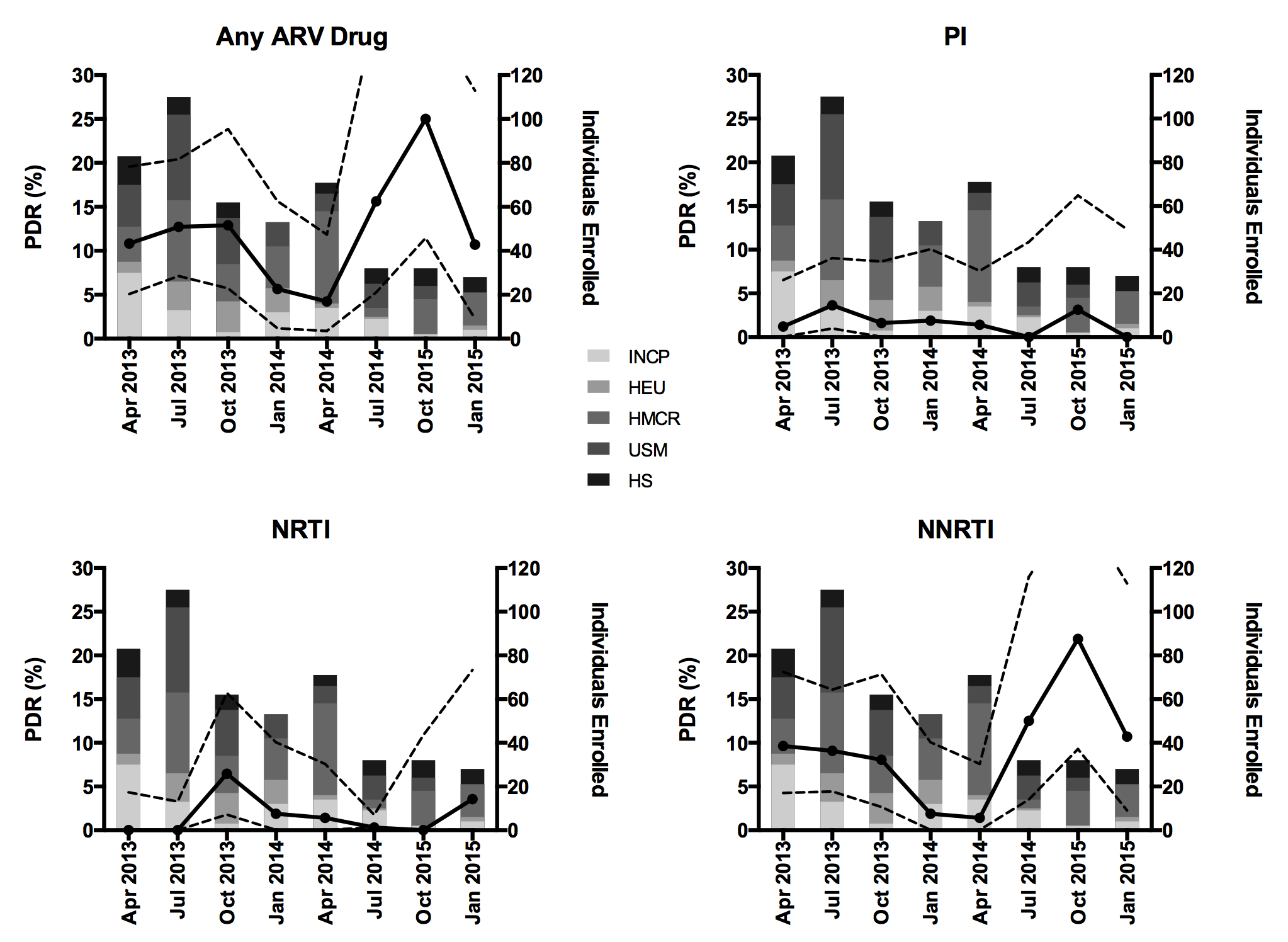

Supplement: S1 Fig — PDR trends by date of enrolment were estimated using a moving average approach, with 4-month windows, moving by 1-month intervals. PDR prevalence and 95% confidence intervals (CI) are shown. The number of individuals contributed by each participating centre for each time window is also shown. PDR, Pre-antiretroviral Treatment Drug Resistance; NRTI, Nucleoside RT Inhibitors; NNRTI, Non-nucleoside RT Inhibitors; PI, protease inhibitors; INCP, Instituto Nacional Cardio Pulmonar (Tegucigalpa); HEU, Hospital Escuela Universitario (Tegucigalpa); HMCR, Hospital Mario Catarino Rivas (San Pedro Sula); USM, Unidad de Salud Metropolitana (La Ceiba); HS, Hospital del Sur (Choluteca). (TIFF) [file pone.0142604.s002.tiff]
