# Supplementary material for: HIV Drug Resistance Surveillance in Honduras after a Decade of Widespread Antiretroviral Therapy
Source: PLoS One. 2015 Nov 11;10(11):e0142604. doi: 10.1371/journal.pone.0142604 (PMC4641727; doi:10.1371/journal.pone.0142604)
Supplement: S1 Table — (DOC) [file pone.0142604.s003.doc]

Table S1. PDR in a Honduran HIV-1-infected cohort by year: 2013-2015

| **WHO Mutation List ^a^** |  | **Complete cohort**  **(April 2013-Aprill 2015)**  **(n=365)** | | | |  | **April 2013-March 2014**  **(n=189)** | | | |  | **April 2014-April 2015**  **(n=176)** | | | | |  | **p value ^c^** |
| --- | --- | --- | --- | --- | --- | --- | --- | --- | --- | --- | --- | --- | --- | --- | --- | --- | --- | --- |
|  |  | n | (%) | [95% CI] | |  | n | (%) | [95% CI] | |  | n | (%) | [95% CI] | | |  |
| Any ARV Drug |  | 42 | (11.5) | [8.4, | 15.2] |  | 24 | (12.7) | [8.3, | 18.3] |  | 18 | (10.2) | [6.2, | 15.7] | |  | NS |
| NNRTI |  | 30 | (8.2) | [5.6, | 11.5] |  | 17 | (9.0) | [5.3, | 14.0] |  | 13 | (7.4) | [4.0, | 12.3] | |  | NS |
| NRTI |  | 8 | (2.2) | [1.0, | 4.3] |  | 4 | (2.1) | [0.6, | 5.3] |  | 4 | (2.3) | [0.6, | 5.7] | |  | NS |
| PI |  | 7 | (1.9) | [0.8, | 3.9] |  | 5 | (2.6) | [0.9, | 6.1] |  | 2 | (1.1) | [0.1, | 4.0] | |  | NS |
| **Stanford Score≥15 ^b^** |  | **Complete cohort**  **(April 2013-Aprill 2015)**  **(n=365)** | | | |  | **April 2013-March 2014**  **(n=189)** | | | |  | **April 2014-March 2015**  **(n=176)** | | | | |  | **p value ^c^** |
|  |  | n | (%) | [95% CI] | |  | n | (%) | [95% CI] | |  | n | (%) | [95% CI] | | |  |
| Any ARV Drug |  | 60 | (16.4) | [12.8, | 20.6] |  | 33 | (17.5) | [12.3, | 23.6] |  | 27 | (15.3) | [10.4, | | 21.5] |  | NS |
| NNRTI |  | 47 | (12.9) | [9.6, | 16.8] |  | 25 | (13.2) | [8.7, | 18.9] |  | 22 | (12.5) | [8.0, | | 18.3] |  | NS |
| NRTI |  | 6 | (1.6) | [0.6, | 3.5] |  | 4 | (2.1) | [0.6, | 5.3] |  | 2 | (1.1) | [0.1, | | 4.0] |  | NS |
| PI |  | 10 | (2.7) | [1.3, | 5.0] |  | 6 | (3.2) | [1.2, | 6.8] |  | 4 | (2.3) | [0.6, | | 5.7] |  | NS |

^a^ Pre-Antiretroviral Treatment Drug Resistance (PDR) estimated using the WHO HIV transmitted drug resistance surveillance mutation list. ^b^ PDR estimated with the Stanford algorithm (v7.0), with a threshold of ≥15 for at least one antiretroviral drug of the specified class. ^c^ p value estimated for April 2013-March 2014 vs. April 2014-April 2015 (Fisher’s exact test). ARV, Antiretroviral; NNRTI, Non-Nucleoside Reverse Transcriptase Inhibitors; NRTI, Nucleoside Reverse Transcriptase Inhibitors; PI, Protease Inhibitors; NS, Not significant (p>0.05).
