# Supplementary material for: HIV Drug Resistance Surveillance in Honduras after a Decade of Widespread Antiretroviral Therapy
Source: PLoS One. 2015 Nov 11;10(11):e0142604. doi: 10.1371/journal.pone.0142604 (PMC4641727; doi:10.1371/journal.pone.0142604)
Supplement: S2 Table — (DOC) [file pone.0142604.s004.doc]

Table S2. PDR in a Honduran HIV-1-infected cohort in recently infected individuals and according to CD4+ T cell counts

| **WHO Mutation List ^a^** |  | **Individuals with longstanding infection**  **(n=261)** | | | |  | **Recently infected individuals ^c^**  **(n=103)** | | | |  | **>500 CD4+ T cells/μL ^d^**  **(n=85)** | | | |  | **350-500 CD4+ T cells/μL ^d^**  **(n=65)** | | | |  | **<350 CD4+ T cells/μL ^d^**  **(n=213)** | | | | |
| --- | --- | --- | --- | --- | --- | --- | --- | --- | --- | --- | --- | --- | --- | --- | --- | --- | --- | --- | --- | --- | --- | --- | --- | --- | --- | --- |
|  |  | n | (%) | [95% CI] | |  | n | (%) | [95% CI] | |  | n | (%) | [95% CI] | |  | n | (%) | [95% CI] | |  | n | (%) | | [95% CI] | |
| Any ARV Drug |  | 28 | (10.7) | [7.2, | 15.1] |  | 14 | (13.6) | [7.6, | 21.8] |  | 12 | (14.1) | [7.5, | 23.4] |  | 5 | (7.7) | [2.5, | 17.0] |  | 25 | (11.7) | | [7.7, | 16.8] |
| NNRTI |  | 22 | (8.4) | [5.4, | 12.5] |  | 8 | (7.8) | [3.4, | 14.7] |  | 8 | (9.4) | [4.2, | 17.7] |  | 3 | (4.6) | [1.0, | 12.9] |  | 19 | (8.9) | | [5.5, | 13.6] |
| NRTI |  | 5 | (1.9) | [0.6, | 4.4] |  | 3 | (2.9) | [0.6, | 8.3] |  | 2 | (2.4) | [0.3, | 8.2] |  | 1 | (1.5) | [0.4, | 8.3] |  | 5 | (2.3) | | [0.8, | 5.4] |
| PI |  | 3 | (1.1) | [0.2, | 3.3] |  | 4 | (3.9) | [1.1, | 9.6] |  | 3 | (3.5) | [0.7, | 10.0] |  | 1 | (1.5) | [0.4, | 8.3] |  | 3 | (1.4) | | [0.3, | 4.1] |
| **Stanford Score≥15 ^b^** |  | **Individuals with longstanding infection**  **(n=261)** | | | |  | **Recently infected individuals ^c^**  **(n=103)** | | | |  | **>500 CD4+ T cells/μL ^d^**  **(n=85)** | | | |  | **350-500 CD4+ T cells/μL ^d^**  **(n=65)** | | | |  | **<350 CD4+ T cells/μL ^d^**  **(n=213)** | | | | |
|  |  | n | (%) | [95% CI] | |  | n | (%) | [95% CI] | |  | n | (%) | [95% CI] | |  | n | (%) | [95% CI] | |  | n | (%) | [95% CI] | | |
| Any ARV Drug |  | 39 | (14.9) | [10.8, | 19.9] |  | 21 | (20.4) | [13.1, | 29.5] |  | 15 | (17.6) | [10.2, | 27.4] |  | 13 | (20.0) | [11.1, | 31.8] |  | 32 | (15.0) | [10.5 | | 20.5] |
| NNRTI |  | 31 | (11.9) | [8.2, | 16.4] |  | 16 | (15.5) | [9.1, | 24.0] |  | 13 | (15.3) | [8.4, | 24.7] |  | 10 | (15.4) | [7.6, | 26.5] |  | 24 | (11.3) | [7.4, | | 16.3] |
| NRTI |  | 3 | (1.1) | [0.2, | 3.3] |  | 3 | (2.9) | [0.6, | 8.3] |  | 1 | (1.2) | [0.0, | 6.4] |  | 1 | (1.5) | [0.4, | 8.3] |  | 4 | (1.9) | [0.5, | | 4.7] |
| PI |  | 7 | (2.7) | [1.1, | 5.4] |  | 3 | (2.9) | [0.6, | 8.3] |  | 2 | (2.4) | [0.3, | 8.2] |  | 2 | (3.1) | [0.4, | 10.7] |  | 6 | (2.8) | [1.0, | | 6.0] |

^a^ Pre-Antiretroviral Treatment Drug Resistance (PDR) estimated using the WHO HIV transmitted drug resistance surveillance mutation list. ^b^ PDR estimated with the Stanford algorithm (v7.0), with a threshold of ≥15 for at least one antiretroviral drug of the specified class. ^c^ Recently infected individuals were defined using a previously described multi-assay algorithm including HIV incidence tests as described in Methods. Data for one individual is missing. **^d^** CD4 T cell count data missing for two individuals. ARV, Antiretroviral; NNRTI, Non-Nucleoside Reverse Transcriptase Inhibitors; NRTI, Nucleoside Reverse Transcriptase Inhibitors; PI, Protease Inhibitors.
